# Supplementary material for: Nicotine rebalances NAD+ homeostasis and improves aging-related symptoms in male mice by enhancing NAMPT activity
Source: Nat Commun. 2023 Feb 17;14:900. doi: 10.1038/s41467-023-36543-8 (PMC9935903; doi:10.1038/s41467-023-36543-8)
Supplement: Supplementary file 1 — Supplementary Information [file 41467_2023_36543_MOESM1_ESM.pdf]

Nicotine rebalances NAD^+ homeostasis and improves aging-related symptoms in male mice by enhancing NAMPT activity

Liang Yang ***et al***

Supplementary Information

Supplementary Figure. 1

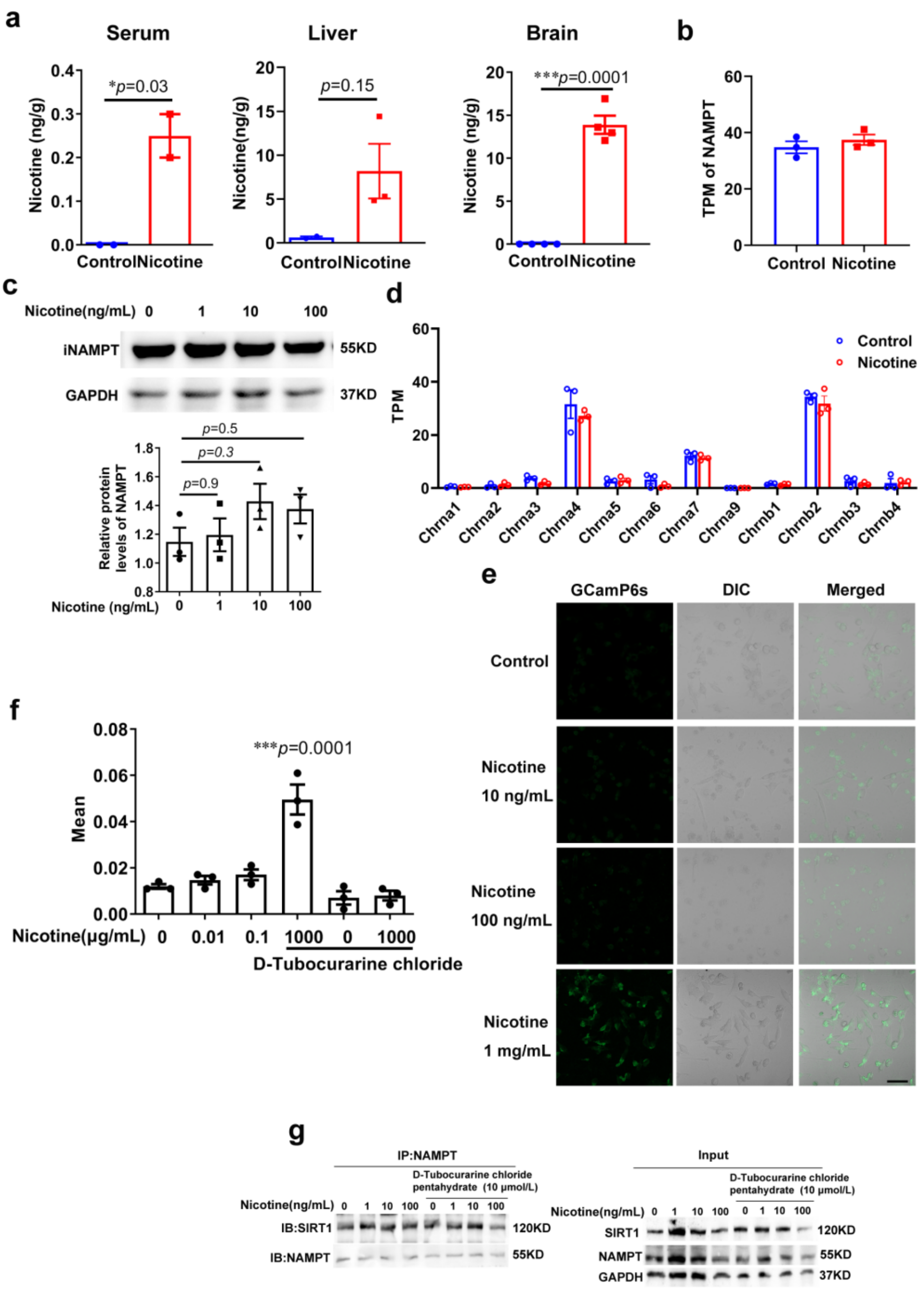

Supplementary Figure 1. Nanogram-trace of Nicotine increased NAD⁺ levels and did not induced the nAchR activation. a. LC-MS and quantification of nicotine (n=2-4) of mice serum, liver and brain with or without nicotine for 6 month. **b.** The gene expression of NAMPT in aged brain with or without nicotine for 6 months. **c.** The protein expression of the NAMPT with nicotine in a dose dependent manner for 48 h. **d.** The gene expression of the nicotinic cholinergic receptor α and β subunits in aged brain with or without nicotine for 6 months. **e.** The images of nicotine effects on the states of nAChRs activity. The scalar bar: 100 μm. **f.** The statistical analysis of the mean fluorescence intensity of Calcium ions in nicotine treatment (n=3 biologically independent samples/group). (**g**) Inhibition of nAChRs activity did not affect the promoting effect of nicotine on SIRT1-NAMPT interaction. Data are means ± SEM. *p* values

were determined by two-sided Student’s t-test (**a,b**), or, One-way ANOVA with Tukey's multiple comparisons test (**c,f**), or two way ANOVA analysis and Fisher’s least significant difference test (**d**).

Supplementary Figure 2.

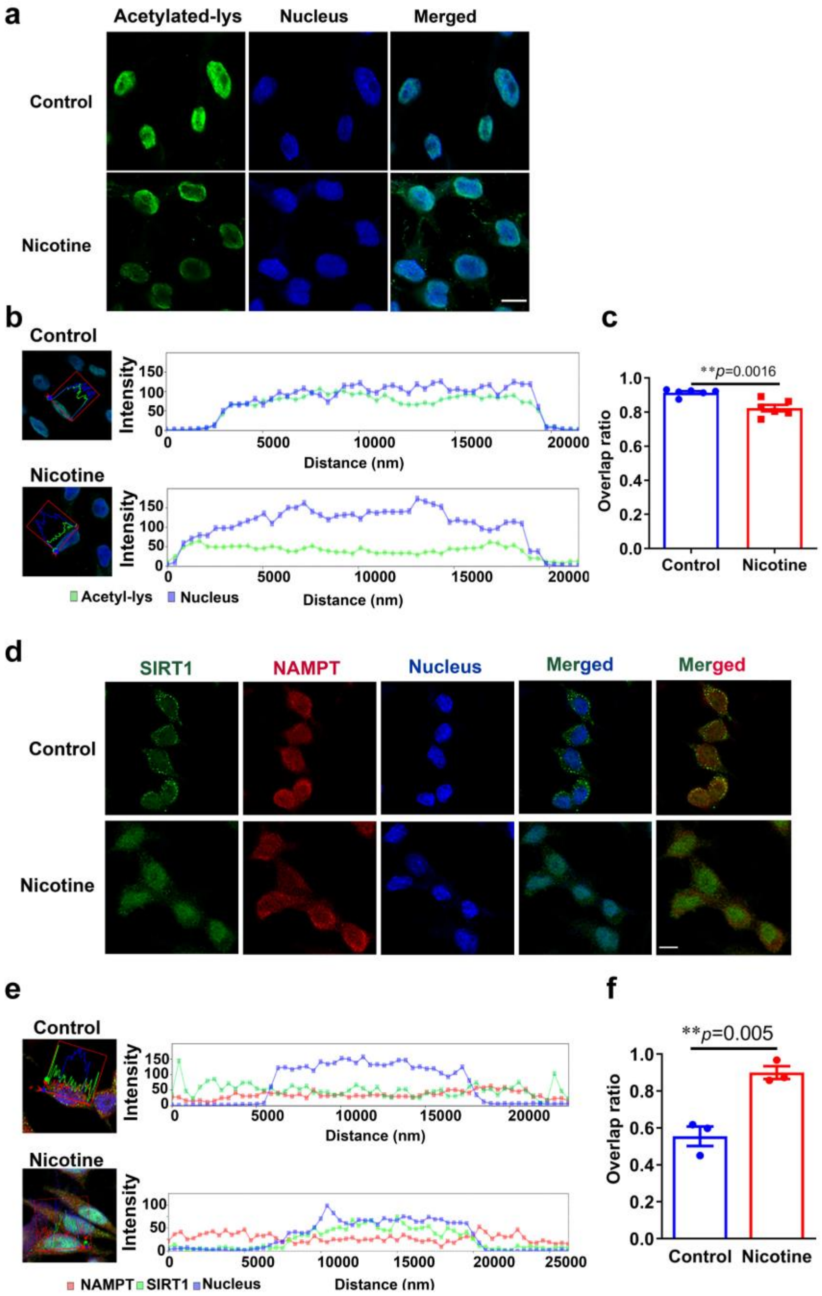

Supplementary Figure 2. Nicotine increased nuclear deacetylation levels and enhanced the co-localization of SIRT1 and NAMPT in HT22 cells. a. Fluorescent images of HT22 cells following the treatment of nicotine effect on nuclear acetylation. The scalar bar: 50 μm. **b.** The fluorescent intensity and co-localization of acetylation levels (Green) and nucleus (Blue) in single cell, administrated with or without nicotine. The scalar bar: 20 μm. **c.** The overlap ratio of acetylation levels and nucleus in HT22 cells (n=6 independent photos/group). **d.** The fluorescent intensity of SIRT1 (Green), NAMPT (Red) and Nucleus (Blue) in single cell. The scalar bar: 50 μm. **e.** The mean fluorescence intensity and

co-localization of SIRT1, NAMPT and Nucleus of HT22 cells administrated with or without nicotine. The scalar bar: 20 μm. **f.** The overlap ratio among SIRT1, NAMPT and nucleus in HT22 cells (n=3 independent photos/group). Data are means ± SEM. *p* values were determined by two-sided Student's t-test (**c, f**).

Supplementary Figure 3.

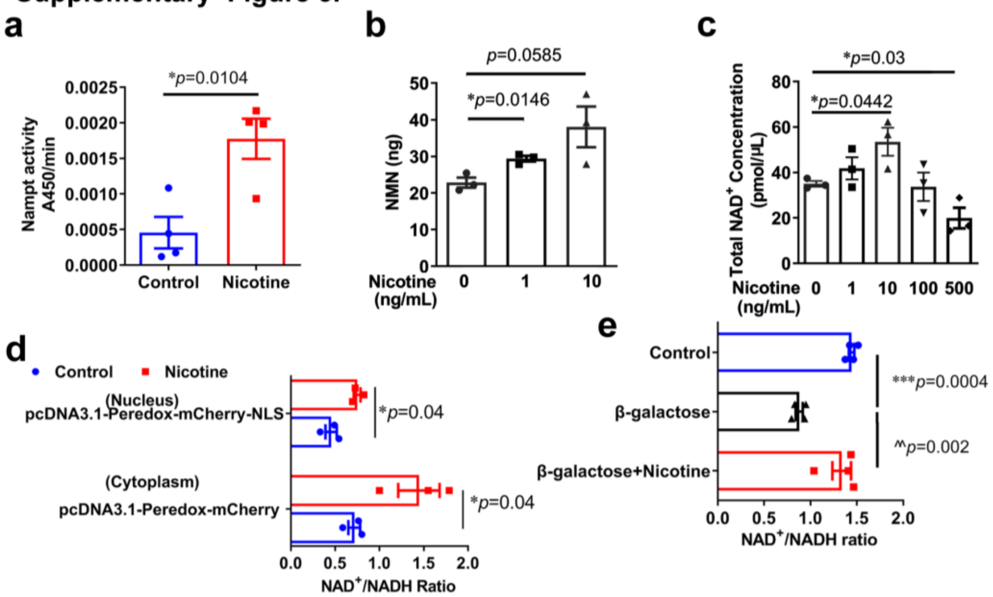

Supplementary Figure 3. Nicotine increases NAD^+ generation and NAMPT activity *in vivo* and *in vitro*. a. NAMPT acute activity in hippocampus of aged mice administered with or without nicotine at 13 months of age (n=4 biologically independent samples/group). **b.** LC-MS and quantification of β-NMN content of HT22 cells with 1 ng/mL nicotine (n=3 biologically independent samples/group) and 10 ng/mL nicotine (n=3) for 48 h. **c.** Total NAD^+ levels of HT22 cells after treated with nicotine at 1 ng/mL and 500 ng/mL for 48 h(n=3 biologically independent samples/group). **d.** The cytosolic NAD^+/NADH ratio (n=3) and nuclear NAD^+/NADH ratio (n=3 biologically independent samples/group) were reflected by biosensors in HT22 cells with or without nicotine. **e.** The NAD^+/NADH ratio of β-galactose-induced aged HT22 cells without nicotine and with nicotine for 7 days (n=4 biologically independent samples/group). Data are means ± SEM. *p* values were determined by two-sided Student's t-test (**a**) or One-way ANOVA with Tukey's multiple comparisons test (**b, c, e**) two way ANOVA analysis and Fisher's least significant difference test (**d**).

Supplementary Figure.4

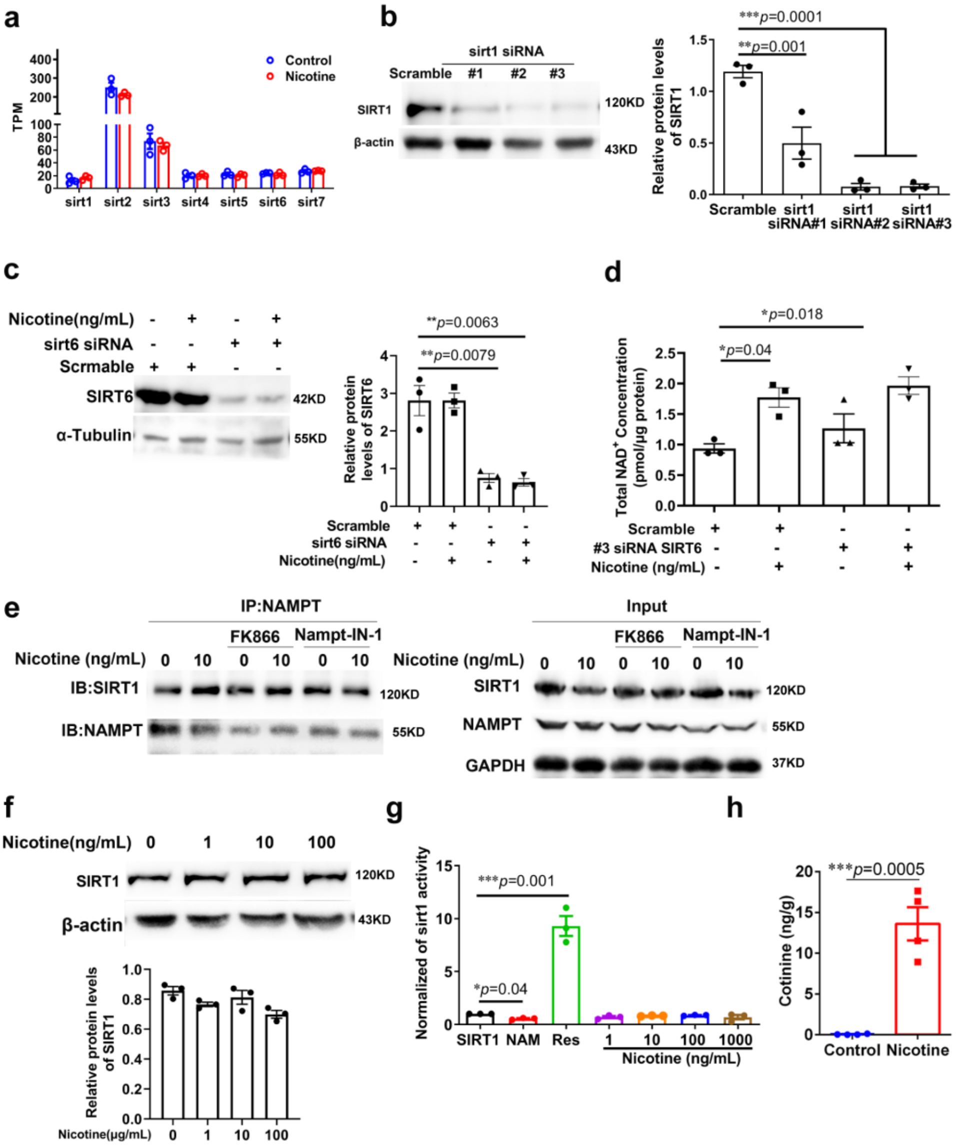

Supplementary Figure 4.Nicotine did not change the SIRT1 expression and not directly affect SIRT1 activity. a. Gene expression of sirt family in aged brain with or without nicotine for 6 months. **b.** Western blot and quantification of the protein expression of SIRT1 in nicotine-treated HT22 cells (n=3 biologically independent samples/group). **c.** Western blot and quantification of the protein expression of SIRT6 in nicotine-treated HT22 cells (n=3 biologically independent samples/group). **d.** The total NAD^+ levels of SIRT6 knockdown cells after nicotine treatment (n=3 biologically independent samples/group). **e.** The effects of NAMPT inhibitor: FK866 and Nampt-IN-1 on SIRT1-NAMPT interaction after nicotine treatment on HT22 cells. **f.** Western blot and quantification of silencing of SIRT1 expression in HT22 cells. **g.** Nicotine has no effect on the purified protein SIRT1 activity (n=3 biologically independent samples/group). **h**. LC-MS and quantification of cotinine content of mice brain with or without nicotine for 6 month (n=4). Data are means ± SEM. *p* values were determined by two way ANOVA analysis and Fisher's least significant difference test (**a**), or two-sided Student's t-test (**h**),or One-way ANOVA with Tukey's multiple comparisons test (**b,c,d,f,g**).

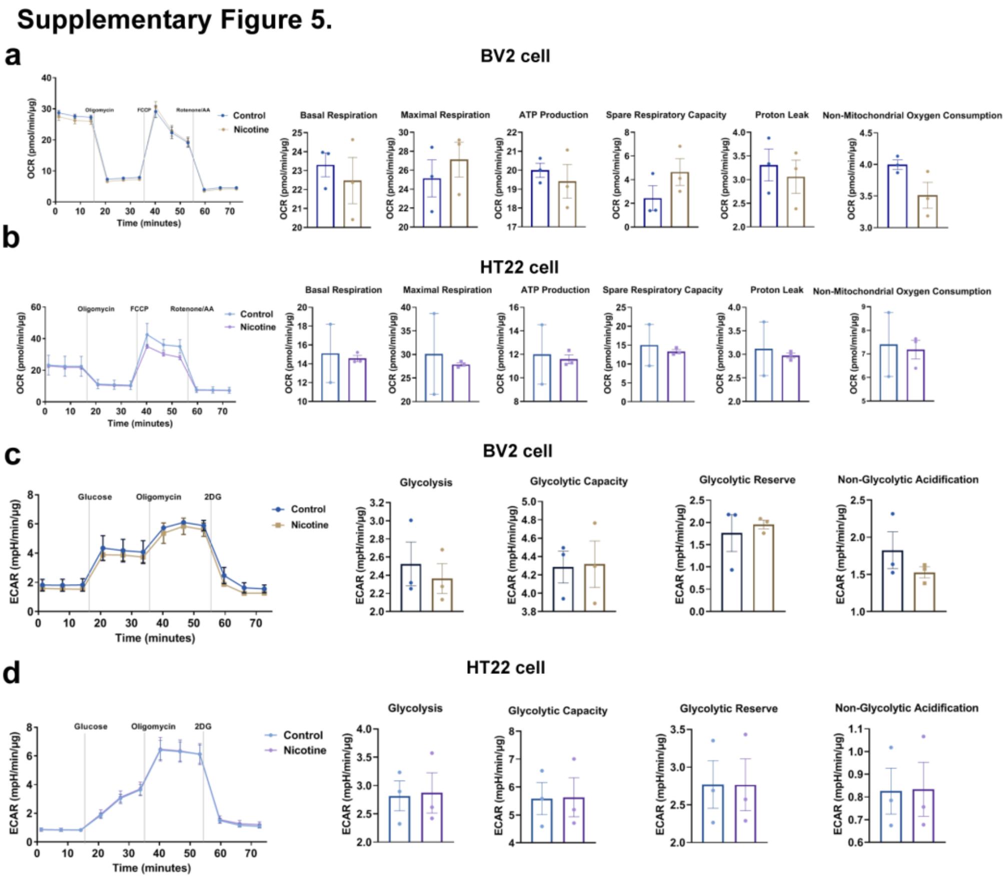

Supplementary Figure 5. Nicotine did not change the OCR and ECAR in BV2, HT22 cells. OCR measurement of nicotine treated **a.** BV2 cell, **b.** HT22 cell and quantitative analysis of Basal respiration, ATP-linked respiration, Proton leak, Maximal respiration, Spare respiratory capacity, Non-mitochondrial oxygen consumption. ECAR of nicotine treated **c.** BV2 cell, **d.** HT22 cell and quantitative analysis of Glycolysis, Glycolytic capacity, Glycolytic reserve and non-glycolytic acidification. Data are means ± SEM (n=2-3 biologically independent samples/group). *p* values were determined by two-sided Student's t-test.

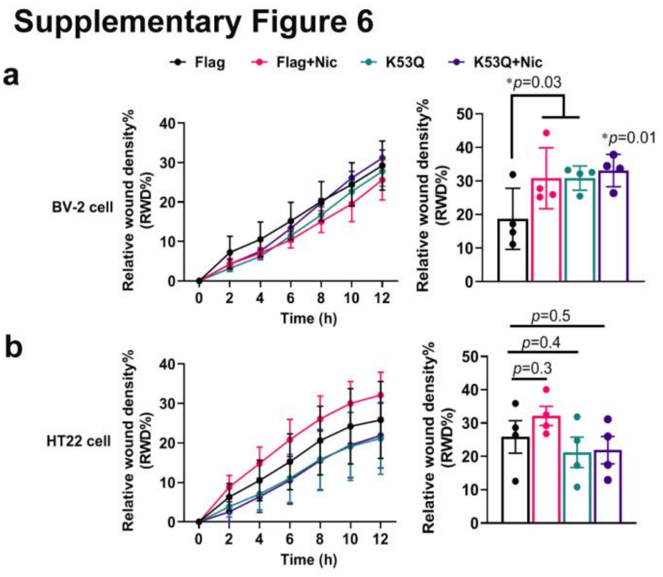

Supplementary Figure 6. Scratch Wound analysis of nicotine on NAMPT-K53Q cells. a. The RWD%

of BV2 cells transfected NAMPT-Flag and NAMPT-K53Q with or without nicotine for 12 h (n=4 biologically independent samples/group).**b.** The RWD% of HT22 cells transfected NAMPT-Flag and NAMPT-K53Q with or without nicotine for 12 h (n=4 biologically independent samples/group). Data are means ± SEM. *p<0.05* values were determined by One-way ANOVA with Tukey's multiple comparisons test (**a, b**) *p Flag+Nicotine vs Flag.

Supplementary Figure 7.

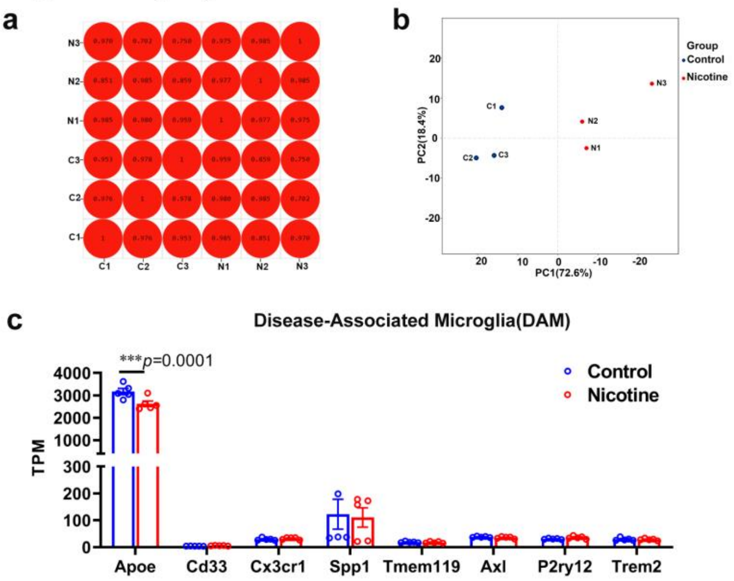

Supplementary Figure **7.The transcriptomic analyses of nicotine administration aged mice brain**. **a.** Spearman's rank correlation of aged mice brain with or without nicotine for 6 months (n=3 biologically independent samples/group). **b.** Principal component analyses showed the percentage of explained variance of aged mice with or without nicotine for 6 months (n=3 biologically independent samples/group). **c.** The TPM of disease-associated microglia genes in aged mice brain with or without nicotine for 6 months (n=4 biologically independent samples/group). Data are means ± SEM. p values were determined by two-sided Student's t-test (**c**).

Supplementary Figure 8.

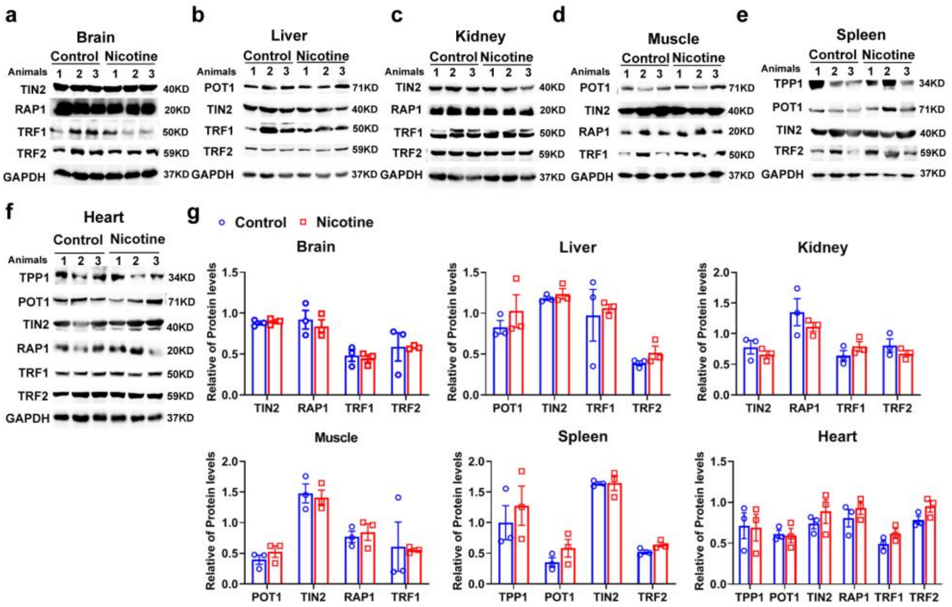

Supplementary Figure 8. Nicotine rescued the telomere shelterin complex in the aged mice. Western blot and quantification of the telomere shelterin complex of **a.** brain, **b.** liver, **c.** kidney, **d.** muscle **e.** spleen and **f.** Heart after 6 months nicotine administration (n=3). g. is a quantification of brain, liver, kidney, muscle, spleen and heart. Quantification is normalized to GAPDH. Data are means ± SEM. *p* values were determined by two way ANOVA analysis and Fisher's least significant difference (**g**).

Supplementary Figure 9.

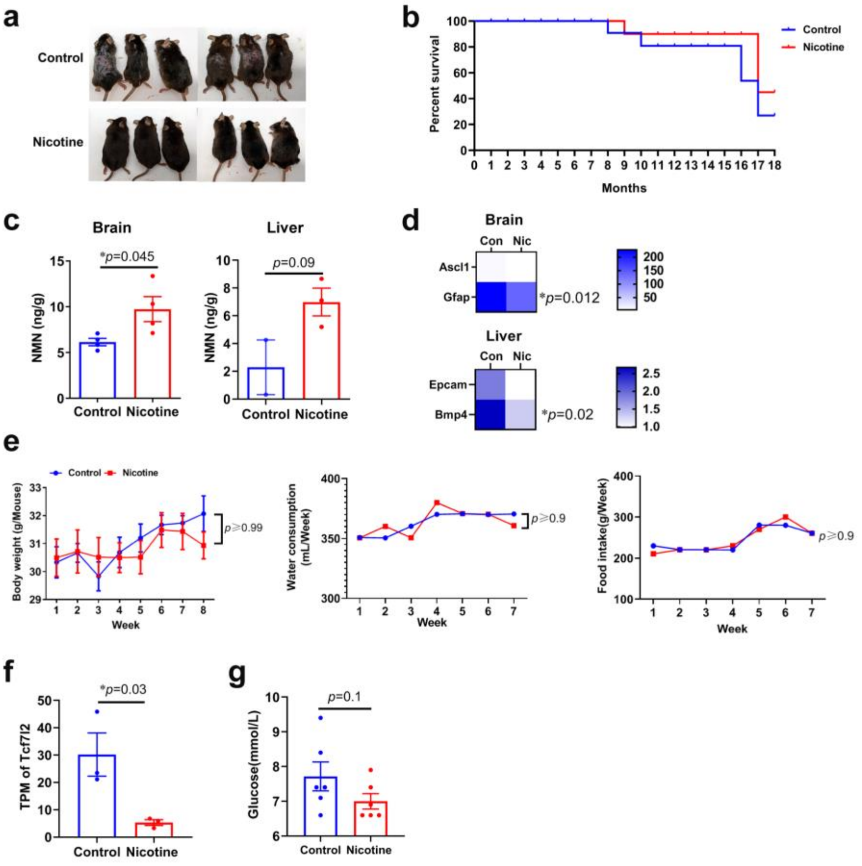

Supplementary Figure 9. Effect of nicotine on anxiety, tumorigenesis, blood glucose, body weight, food and water consumption. a. The representative images of aged male mice drink with nicotine from 7 to 13-month-old mice (n=12 biologically independent samples/group). The mouse images were taken after 6 months of nicotine treatment. **b.** Kaplan-Meier curves of male C57BL/6J mice (control group n=24, Nicotine group n=24 biologically independent samples/group)**. c.** LC-MS and quantification of β-NMN content in brain of aged mice administered with or without nicotine at 13 months of age (n=2-3 biologically independent samples/group). LC-MS and quantification of β-NMN content in liver of aged mice administered with or without nicotine at 13 months of age (n=2-3 biologically independent samples/group) **d.** Heatmap of the TPM of tumorigenesis markers in brain: Ascl1 and Gfap (n=3 biologically independent samples/group) and liver: Epcam and Bmp4 (n=3 biologically independent samples/group) of mice with nicotine administration for 6 months, *p* value Control (Con) vs Nicotine (Nic).**e.** The body weight, food and water consumption of control and nicotine group in 7 weeks (n=6 biologically independent samples/group). **f.** TPM of brain *Tcf7l2* gene in aged mice with nicotine for 6 months (n=3 biologically independent samples/group).**g.** the blood glucose of aged mice with nicotine administration for 2 months (n=6 biologically independent samples/group). Data are means ± SEM. *p* values were determined by, two-sided Student's t-test (**c, d, e, f, g**).

Supplementary Table 1 Antibodies and compounds commercial information

REAGENT or RESOURCE	SOURCE	IDENTIFIER
**Antibody**		
Rabbit Monoclonal anti-SIRT1(D1D7)	Cell Signaling Technology	Cat#9475T;RRID: AB_2617130
Mouse Monoclonal anti-PBEF(E-3)	Santa Cruz Biotechnology	Cat#sc393444;RRID:AB_2894708
Rabbit Polyclonal anti-GAPDH	Abcam	Cat#ab9485; RRID: AB_307275
Mouse Monoclonal anti-β-actin	Sigma-Aldrich	Cat#A1978; RRID: AB_476692
Rabbit Monoclonal anti NF-κb(D14E12)	Cell Signaling Technology	Cat#8242;RRID:AB_10859369
Rabbit Monoclonal anti-SIRT6(D8D12)	Cell Signaling Technology	Cat#12486;RRID:AB_2636969
Mouse Monoclonal anti-p53	Santa Cruz Biotechnology	Cat# sc71819;RRID:AB_1126979
Mouse Monoclonal anti-SIRT1	Santa Cruz Biotechnology	Cat# sc74465; RRID:AB_1129462
Mouse Monoclonal anti-PGC-1α(D-5)	s	Cat#sc518025;RRID:AB_2890187
Rabbit Monoclonal anti-BDNF	Cell Signaling Technology	Cat# 47808;RRID:AB_2894709
Rabbit Monoclonal anti-Doublecortin	Abcam	Cat#ab207175;RRID:AB_2894710
Rabbit Polyclonal Anti-POT1	ABclonal	Cat# A1491; RRID:AB_2761791
Rabbit Polyclonal Anti-TPP1	ABclonal	Cat#A5627;RRID:AB_2766387
Rabbit Polyclonal Anti-Rap1A	ABclonal	Cat#A0975;RRID: AB_2757494
Rabbit Polyclonal Anti-TERF1	ABclonal	Cat#A0137;RRID: AB_2766105
Rabbit Polyclonal Anti-TERF2	ABclonal	Cat#A16316;RRID:AB_2772562
Rabbit Polyclonal Anti-TIN2/TINF2	ABclonal	Cat#A9750;RRID:AB_2767352
Mouse Monoclonal AC-lysine(AKL5C1)	Santa Cruz Biotechnology	Cat#SC-32268
Goat anti-MOUSE IgG (H+L)	Jackson immune research	Cat# 223-005-024
Goat anti-Rabbit IgG (H+L)	Jackson immune research	Cat# 323-005-021

Alexa 488-conjugated Goat anti-Rabbit IgG antibody	Thermo Scientific	Cat#A32731
Alexa 555-conjugated Goat anti-Mouse IgG antibody	Thermo Scientific	Cat#A32727

**Chemicals**		
DAPI	Sigma-Aldrich	Cat#D9542
Nicotine	Sigma-Aldrich	Cat#N3876
β-NMN	Sigma-Aldrich	Cat#N3501
Resveratrol	MedChemExpress	Cat#HY-16561
SRT1720	MedChemExpress	Cat#HY-10532
FK866	MedChemExpress	Cat#HY-50876
Nampt-IN-1	MedChemExpress	Cat# HY-12971
cotinine	MedChemExpress	Cat#HY-B1178
Selisistat	MedChemExpress	Cat#HY15452
Phosphatase inhibitor cocktail	Roche	Cat#5892791001
Protein A+G Agarose beads	Santa Cruz Biotechnology	Cat#sc-2003
D-galactose	MedChemExpress	Cat#HY-N0210
F18-FDG	Gosun cyclotron Medicine	
D-Tubocurarine chloride pentahydrate	MedChemExpress	CAT#HY-125901
**Critical Commercial Assays**		
CycLex® NAMPT Colorimetric Assay Kit Ver.2	MBL	Cat# CY-1251V2
NAD/NADH assay kit	Abcam	Cat# ab65348
Pierce™ BCA Protein Assay Kit	Thermo Scientific	Cat# 23227
Protein Carbonyl Colorimetric Assay Kit	Cayman	Cat# 10005020-96
Universal SIRT Activity Assay Kit	abcam	Cat# ab156915
SIRT1 activity assay kit	Sigma-Aldrich	Cat#
Hifair® Ⅱ 1st Strand cDNA Synthesis Kit (gDNA digester plus)	Yeasen	Cat# 11121ES60
Hifair® Ⅲ One Step	Yeasen	Cat# 11143ES50

RT-qPCR SYBR Green Kit		
Total Antioxidant Capacity Assay Kit	Beyotime	Cat#S0119
Cu/Zn-SOD and Mn-SOD Assay Kit	Beyotime	Cat#S0103
Cell Mito Stress Test Kit	Agilent Technologies	Cat#103015-100
Glycolysis stress Test Kit	Agilent Technologies	Cat#103020-100
**Experimental Models: Cell Lines**		
HT22 cell line	BeNaCultureCollection	Cat# BNCC337709
BV2 cell line	BeNaCultureCollection	Cat# BNCC337749
**Experimental Models:Organisms/Strains**		
Mouse: C57bl/6J	Guangdong Medical Laboratory Animal Center	
**Software and Algorithms**		
GraphPad Prism	GraphPad Software (version8)	http://www.graphpad.com/scientificsoftware/prism/
ImageJ	NIH	http://imagej.nih.gov/ij/
Zen 2011	Carl Zeiss	https://www.zeiss.com/microscopy/int/downloads.html
Noldus	Noldus	https://www.noldus.com.cn/animal-behavior-research/
Quantity One	Biored	https://www.bio-rad.com
Incucyte ®S3	Sartorius	www.sartorius.com/Incucyte
Wave	Agilent Technologies	www.agilent.com

Supplementary Table 2 siRNA and Oligonucleotides

siRNA	
Scrambled:FW:5'- UUCUUCGAACGUGUCACGUTT-3'	GenePharma
Scrambled:RV:5'- ACGUGACACGUUCGGAGAATT -3'	GenePharma

#1 sirt1:FW: 5’-GCGGAUAGGUCCAUAUACUTT-3’	GenePharma
#1 sirt1:RV:5’- AGUAUAUGGACCUAUCCGCTT-3’	GenePharma
#2 sirt1:FW: 5’- CCGUCUCUGUGUCACAAAUTT-3’	GenePharma
#2 sirt1:RV: 5’- AUUUGUGACACAGAGACGGTT-3’	GenePharma
#3 sirt1:FW:5’- GGGAUCAAGAGGUUGUUAATT-3’	GenePharma
#3 sirt1:RV:5’- UUAACAACCUCUUGAUCCCTT-3’	GenePharma
#1 sirt6:FW: 5’- GGUCAUUGUCAACCUGCAATT-3’	GenePharma
#1 sirt6:RV:5’- UUGCAGGUUGACAAUGACCTT-3’	GenePharma
#2 sirt6:FW: 5’- GCUGCACGGAAACAUGUUUTT-3’	GenePharma
#2 sirt6:RV: 5’- AAACAUGUUUCCGUGCAGCTT-3’	GenePharma
#3 sirt6:FW:5’- GCUACGUGGAUGAGGUGAUTT-3’	GenePharma
#3 sirt6:RV:5’- AUCACCUCAUCCACGUAGCTT-3’	GenePharma
Oligonucleotides	
Telomeric:FW:5’- CGGTTTGTTTGGGTTTGGGTTTGGGTTTGGGTTTGGGTT-3’	Callicott and Womack, 2006; Cawthon, 2002)
Telomeric:RV:5’- GGCTTGCCTTACCCTTACCCTTACCCTTACCCTTACCCT-3’	Callicott and Womack, 2006; Cawthon, 2002)
36B4:FW:5’- ACTGGTCTAGGACCCGAGAAG-3’	Callicott and Womack, 2006; Cawthon, 2002)
36B4:RV:5’- TCAATGGTGCCTCTGGAGATT-3’	Callicott and Womack, 2006; Cawthon, 2002)
