# Supplementary material for: Nicotine rebalances NAD+ homeostasis and improves aging-related symptoms in male mice by enhancing NAMPT activity
Source: Nat Commun. 2023 Feb 17;14:900. doi: 10.1038/s41467-023-36543-8 (PMC9935903; doi:10.1038/s41467-023-36543-8)
Supplement: Supplementary file 3 — Reporting Summary [file 41467_2023_36543_MOESM3_ESM.pdf]

nature portfolio

Corresponding author(s): Xiang LI

Last updated by author(s): Jan 15, 2023

Reporting Summary

Nature Portfolio wishes to improve the reproducibility of the work that we publish. This form provides structure for consistency and transparency in reporting. For further information on Nature Portfolio policies, see our Editorial Policies and the Editorial Policy Checklist.

Statistics

For all statistical analyses, confirm that the following items are present in the figure legend, table legend, main text, or Methods section.

n/a	Confirmed	
☐	☒	The exact sample size (n) for each experimental group/condition, given as a discrete number and unit of measurement
☐	☒	A statement on whether measurements were taken from distinct samples or whether the same sample was measured repeatedly
☐	☒	The statistical test(s) used AND whether they are one- or two-sided<br>*Only common tests should be described solely by name; describe more complex techniques in the Methods section.*
☐	☒	A description of all covariates tested
☐	☒	A description of any assumptions or corrections, such as tests of normality and adjustment for multiple comparisons
☐	☒	A full description of the statistical parameters including central tendency (e.g. means) or other basic estimates (e.g. regression coefficient) AND variation (e.g. standard deviation) or associated estimates of uncertainty (e.g. confidence intervals)
☒	☐	For null hypothesis testing, the test statistic (e.g. F, t, r) with confidence intervals, effect sizes, degrees of freedom and P value noted<br>*Give P values as exact values whenever suitable.*
☒	☐	For Bayesian analysis, information on the choice of priors and Markov chain Monte Carlo settings
☒	☐	For hierarchical and complex designs, identification of the appropriate level for tests and full reporting of outcomes
☒	☐	Estimates of effect sizes (e.g. Cohen's d, Pearson's r), indicating how they were calculated

Our web collection on statistics for biologists contains articles on many of the points above.

Software and code

Policy information about availability of computer code

Data collection	Data collection described in Methods section. No custom software was used.
Data analysis	Data analysis is described in the Methods section. Western blot images and micrograph images were analysis by Image J and GraphPad Prism 8 and assembled in Adobe Photoshop CC8 2018,qRT-PCR data was analyzed in Graphpad 8.0 . the LC-MS/MS data acquisition and processing was done using the Analyst software (AB Sciex, USA) version. The PET images were analyzed in Amide software.Cell energy metabolism was detected and analyzed using Agilent Wave desktop v2.6.Cell scratch wound assay and analysis were performed using Incucyte2021A

For manuscripts utilizing custom algorithms or software that are central to the research but not yet described in published literature, software must be made available to editors and reviewers. We strongly encourage code deposition in a community repository (e.g. GitHub). See the Nature Portfolio guidelines for submitting code & software for further information.

Data

Policy information about availability of data

All manuscripts must include a data availability statement. This statement should provide the following information, where applicable:

- Accession codes, unique identifiers, or web links for publicly available datasets
- A description of any restrictions on data availability
- For clinical datasets or third party data, please ensure that the statement adheres to our policy

Data supporting the findings of this study are available within the article and its Supplementary Figures.The source data underlying Figs. 1-3,5-10 and

Supplementary Figs.1-9 are provided as a Source Data file. Additional details on datasets and protocols that support the findings of this study will be made available by the corresponding author upon reasonable request.

Human research participants

Policy information about studies involving human research participants and Sex and Gender in Research.

Reporting on sex and gender	N/A
Population characteristics	N/A
Recruitment	N/A
Ethics oversight	N/A

Note that full information on the approval of the study protocol must also be provided in the manuscript.

Field-specific reporting

Please select the one below that is the best fit for your research. If you are not sure, read the appropriate sections before making your selection.

☒ Life sciences ☐ Behavioural & social sciences ☐ Ecological, evolutionary & environmental sciences

For a reference copy of the document with all sections, see nature.com/documents/nr-reporting-summary-flat.pdf

Life sciences study design

All studies must disclose on these points even when the disclosure is negative.

Sample size	In general, no calculations were done to determine sample size. Sample size was determined based on standards for experimental cell biology and animal studies, attempting to have a minimum of N = 3 biological replicates with sufficient reproducibility. Cell cultures were maintained and studied, while providing enough material for protein and mRNA expression levels analysis. The activity assay and RT-PCR include technical replicates per biological replicate. In experiments involving animals, N≥3 with further behavioural experimental details on sample . In experiments involving PET,LC-MS,(brain, plasma) ,N=3 are included in the Methods section.
Data exclusions	No data were excluded
Replication	All experimental findings were replicated at least 3 times with enough reproducibility. Attempts of data replication were, therefore, successful.
Randomization	Samples were allocated randomly for culture and analysis. For animal studies, allocation into experimental groups is not relevant because, in this study, we only describe proof-of-concept testing of nicotine in more than 3 animals, and measure drug brain/liver concentration and plasma levels.
Blinding	In general, the investigators were blind at the time of experiment execution and data acquisition. the PET and LCM data was captured by the technicians who were blind to group/sample allocation since samples were identified only by individual numbering without subdivision into groups. Investigators were blinded to the group allocation during the data collection and blinded to sample identity for the analysis of immunohistochemistry and western blotting.

Behavioural & social sciences study design

All studies must disclose on these points even when the disclosure is negative.

Study description	*Briefly describe the study type including whether data are quantitative, qualitative, or mixed-methods (e.g. qualitative cross-sectional, quantitative experimental, mixed-methods case study).*
Research sample	*State the research sample (e.g. Harvard university undergraduates, villagers in rural India) and provide relevant demographic information (e.g. age, sex) and indicate whether the sample is representative. Provide a rationale for the study sample chosen. For studies involving existing datasets, please describe the dataset and source.*
Sampling strategy	*Describe the sampling procedure (e.g. random, snowball, stratified, convenience). Describe the statistical methods that were used to predetermine sample size OR if no sample-size calculation was performed, describe how sample sizes were chosen and provide a rationale for why these sample sizes are sufficient. For qualitative data, please indicate whether data saturation was considered, and what criteria were used to decide that no further sampling was needed.*

Data collection	*Provide details about the data collection procedure, including the instruments or devices used to record the data (e.g. pen and paper, computer, eye tracker, video or audio equipment) whether anyone was present besides the participant(s) and the researcher, and whether the researcher was blind to experimental condition and/or the study hypothesis during data collection.*
Timing	*Indicate the start and stop dates of data collection. If there is a gap between collection periods, state the dates for each sample cohort.*
Data exclusions	*If no data were excluded from the analyses, state so OR if data were excluded, provide the exact number of exclusions and the rationale behind them, indicating whether exclusion criteria were pre-established.*
Non-participation	*State how many participants dropped out/declined participation and the reason(s) given OR provide response rate OR state that no participants dropped out/declined participation.*
Randomization	*If participants were not allocated into experimental groups, state so OR describe how participants were allocated to groups, and if allocation was not random, describe how covariates were controlled.*

Ecological, evolutionary & environmental sciences study design

All studies must disclose on these points even when the disclosure is negative.

Study description	*Briefly describe the study. For quantitative data include treatment factors and interactions, design structure (e.g. factorial, nested, hierarchical), nature and number of experimental units and replicates.*
Research sample	*Describe the research sample (e.g. a group of tagged Passer domesticus, all Stenocereus thurberi within Organ Pipe Cactus National Monument), and provide a rationale for the sample choice. When relevant, describe the organism taxa, source, sex, age range and any manipulations. State what population the sample is meant to represent when applicable. For studies involving existing datasets, describe the data and its source.*
Sampling strategy	*Note the sampling procedure. Describe the statistical methods that were used to predetermine sample size OR if no sample-size calculation was performed, describe how sample sizes were chosen and provide a rationale for why these sample sizes are sufficient.*
Data collection	*Describe the data collection procedure, including who recorded the data and how.*
Timing and spatial scale	*Indicate the start and stop dates of data collection, noting the frequency and periodicity of sampling and providing a rationale for these choices. If there is a gap between collection periods, state the dates for each sample cohort. Specify the spatial scale from which the data are taken*
Data exclusions	*If no data were excluded from the analyses, state so OR if data were excluded, describe the exclusions and the rationale behind them, indicating whether exclusion criteria were pre-established.*
Reproducibility	*Describe the measures taken to verify the reproducibility of experimental findings. For each experiment, note whether any attempts to repeat the experiment failed OR state that all attempts to repeat the experiment were successful.*
Randomization	*Describe how samples/organisms/participants were allocated into groups. If allocation was not random, describe how covariates were controlled. If this is not relevant to your study, explain why.*
Blinding	*Describe the extent of blinding used during data acquisition and analysis. If blinding was not possible, describe why OR explain why blinding was not relevant to your study.*

Did the study involve field work? ☐ Yes ☐ No

Field work, collection and transport

Field conditions	*Describe the study conditions for field work, providing relevant parameters (e.g. temperature, rainfall).*
Location	*State the location of the sampling or experiment, providing relevant parameters (e.g. latitude and longitude, elevation, water depth).*
Access & import/export	*Describe the efforts you have made to access habitats and to collect and import/export your samples in a responsible manner and in compliance with local, national and international laws, noting any permits that were obtained (give the name of the issuing authority, the date of issue, and any identifying information).*
Disturbance	*Describe any disturbance caused by the study and how it was minimized.*

Reporting for specific materials, systems and methods

We require information from authors about some types of materials, experimental systems and methods used in many studies. Here, indicate whether each material, system or method listed is relevant to your study. If you are not sure if a list item applies to your research, read the appropriate section before selecting a response.

Materials & experimental systems

n/a	Involved in the study
☐	☒ Antibodies
☐	☒ Eukaryotic cell lines
☒	☐ Palaeontology and archaeology
☐	☒ Animals and other organisms
☒	☐ Clinical data
☒	☐ Dual use research of concern

Methods

n/a	Involved in the study
☒	☐ ChIP-seq
☒	☐ Flow cytometry
☒	☐ MRI-based neuroimaging

Antibodies

Antibodies used

1.Rabbit Monoclonal anti-SIRT1(D1D7) Cell Signaling TechnologyCat#9475T;RRID: AB_2617130 Dilution: Immunofluorescence1:200
2.Mouse Monoclonal anti-PBEF(E-3) Santa Cruz Biotechnology Cat#sc393444;RRID:AB_2894708 Dilution: Western blot: 1:1000, immunocoprecipitation:1 μg/500 μg protein
3.Rabbit polyclonal anti-GAPDH Abcam Cat#ab9485; RRID: AB_307275 Dilution: Western blot: 1:10000
4.Mouse monoclonal anti-β-actin Sigma-Aldrich Cat#A1978; RRID: AB_476692 Dilution: Western blot: 1:10000
5.Rabbit Monoclonal anti NF-κb(D14E12) CellSignaling Technology Cat#8242;RRID:AB_10859369 Dilution: Western blot: 1:1000
6.Rabbit Monoclonal anti-SIRT6(D8D12) CellSignaling Technology Cat#12486;RRID:AB_2636969 Dilution: Western blot: 1:1000
7.Mouse monoclonal anti-p53((2B2.71) Santa Cruz Biotechnology Cat# sc71819;RRID:AB_1126979 Dilution: Western blot: 1:1000
8.Mouse monoclonal anti-SIRT1(B-7)Santa Cruz Biotechnology Cat# sc74465; RRID:AB_1129462 Dilution: Western blot: 1:1000, .immunocoprecipitation:1 μg/500 μg protein
9.Mouse monoclonal anti-PGC-1α(D-5) SantaCruzBiotechnology Cat#sc518025;RRID:AB_2890187 Dilution: Western blot: 1:1000.
10.Rabbit Monoclonal anti-BDNF Cell Signaling Technology Cat# 47808;RRID:AB_2894709 Dilution: Western blot: 1:1000.
11.Rabbit Monoclonal anti-Doublecortin Abcam Cat#ab207175;RRID:AB_2894710 Dilution: Western blot: 1:1000, Immunofluorescence1:200.
12.Rabbit Polyclonal Anti-POT1 ABclonal Cat# A1491; RRID:AB_2761791 Dilution: Western blot: 1:1000
13.Rabbit Polyclonal Anti-TPP1 ABclonal Cat#A5627;RRID:AB_2766387 Dilution: Western blot: 1:1000
14.Rabbit Polyclonal Anti-Rap1A ABclonal Cat#A0975;RRID: AB_2757494 Dilution: Western blot: 1:1000
15.Rabbit Polyclonal Anti-TERF1 ABclonal Cat#A0137;RRID: AB_2766105 Dilution: Western blot: 1:1000
16.Rabbit Polyclonal Anti-TERF2 ABclonal Cat#A16316;RRID:AB_2772562 Dilution: Western blot: 1:1000
17.Rabbit Polyclonal Anti-TIN2/TINF2 ABclonal Cat#A9750;RRID:AB_2767352 Dilution: Western blot: 1:1000
18.Mouse monoclonal AC-lysine(AKL5C1) Santa Cruz Biotechnology Cat#sc-32268 Dilution: Western blot: 1:1000, immunocoprecipitation:1 μg/500 μg protein
19.goat anti-MOUSE IgG (H+L) Jackson immune research Cat# 223-005-024 RRID: AB_2339261 Dilution: Western blot: 1:5000
20.goat anti-Rabbit IgG (H+L) Jackson immune research Cat# 323-005-021 RRID: AB_2314648 Dilution: Western blot: 1:5000
21.Alexa 488-conjugated Goat anti-Rabbit IgG antibody Thermo Scientific Cat#A32731
22.Alexa 555-conjugated Goat anti-Mouse IgG antibody Thermo Scientific Cat#A32727

Validation

1.SirT1 (D1D7) Rabbit mAb #9475https://www.cellsignal.com/products/primary-antibodies/sirt1-d1d7-rabbit-mab/9475
2.PBEF(E-3) Mouse sc393444https://www.scbt.com/zh/p/pbef-antibody-e-3
3.anti-GAPDH https://www.abcam.com/gapdh-antibody-loading-control-ab9485.html
4.Mouse monoclonal anti-β-actin https://www.sigmaaldrich.cn/CN/zh/product/sigma/a1978
5.Rabbit Monoclonal anti NF-κb(D14E12) https://www.cellsignal.cn/products/primary-antibodies/nf-kb-p65-d14e12-xp-rabbit-mab/8242
6.Rabbit Monoclonal anti-SIRT6(D8D12) https://www.cellsignal.cn/products/primary-antibodies/sirt6-d8d12-rabbit-mab/12486?_=1671698258820&Ntt=sirt6(d8d12)&tahead=true
7.Mouse monoclonal anti-p53 https://www.scbt.com/p/p53-antibody-2b2-71?requestFrom=search
8.Mouse monoclonal anti-SIRT1 https://www.scbt.com/p/sirt1-antibody-b-7?requestFrom=search
9.Mouse monoclonal anti-PGC-1α(D-5) https://www.scbt.com/p/pgc-1alpha-antibody-d-5?requestFrom=search
10.Rabbit Monoclonal anti-BDNF https://www.cellsignal.cn/products/primary-antibodies/bdnf-antibody/47808?_=1671698291432&Ntt=47808&tahead=true
11.Rabbit Monoclonal anti-Doublecortin https://www.abcam.com/doublecortin-antibody-epr19997-ab207175.html;
12.Rabbit Polyclonal Anti-POT1 https://abclonal.com.cn/catalog/A1491
13.Rabbit Polyclonal Anti-TPP1 https://abclonal.com.cn/catalog/A5627

14.Rabbit Polyclonal Anti-Rap1A https://abclonal.com.cn/catalog/A0975
15.Rabbit Polyclonal Anti-TERF1 https://abclonal.com.cn/catalog/A0137
16.Rabbit Polyclonal Anti-TERF2 https://abclonal.com.cn/catalog/A16316
17.Rabbit Polyclonal Anti-TIN2/TINF2 https://abclonal.com.cn/catalog/A9750
18.Alexa 488-conjugated Goat anti-Rabbit IgG antibody https://www.thermofisher.cn/cn/zh/antibody/product/Goat-anti-Rabbit-IgG-H-L-Highly-Cross-Adsorbed-Secondary-Antibody-Polyclonal/A32731
19.Alexa 555-conjugated Goat anti-Mouse IgG antibody https://www.thermofisher.cn/cn/zh/antibody/product/Goat-anti-Mouse-IgG-H-L-Highly-Cross-Adsorbed-Secondary-Antibody-Polyclonal/A32727
20. Mouse monoclonal AC-lysine(AKL5C1) https://www.scbt.com/zh/p/ac-lysine-antibody-akl5c1
21.goat anti-MOUSE IgG (H+L) Jackson immune research https://www.jacksonimmuno.com/catalog/products/115-005-003
22.goat anti-Rabbit IgG (H+L) Jackson immune researchhttps://www.jacksonimmuno.com/catalog/products/323-005-021
.

Eukaryotic cell lines

Policy information about cell lines and Sex and Gender in Research

Cell line source(s)	HT22 and BV2 cells were obtained from BeNaCultureCollection (Beijing, China)
Authentication	Not authenticated
Mycoplasma contamination	Not tested for Mycoplasma
Commonly misidentified lines (See ICLAC register)	None

Palaeontology and Archaeology

Specimen provenance	*Provide provenance information for specimens and describe permits that were obtained for the work (including the name of the issuing authority, the date of issue, and any identifying information). Permits should encompass collection and, where applicable, export.*
Specimen deposition	*Indicate where the specimens have been deposited to permit free access by other researchers.*
Dating methods	*If new dates are provided, describe how they were obtained (e.g. collection, storage, sample pretreatment and measurement), where they were obtained (i.e. lab name), the calibration program and the protocol for quality assurance OR state that no new dates are provided.*

☐ Tick this box to confirm that the raw and calibrated dates are available in the paper or in Supplementary Information.

Ethics oversight	*Identify the organization(s) that approved or provided guidance on the study protocol, OR state that no ethical approval or guidance was required and explain why not.*

Note that full information on the approval of the study protocol must also be provided in the manuscript.

Animals and other research organisms

Policy information about studies involving animals; ARRIVE guidelines recommended for reporting animal research, and Sex and Gender in Research

Laboratory animals	C57BL/6J mouse age 6-18 Months
Wild animals	No wild animals used.
Reporting on sex	The mice involved in the animal experiment in this paper are all male, in order to avoid the influence of estrogen of female animal on the experiment.
Field-collected samples	This study did not involve samples collected from the field
Ethics oversight	All animals C57BL/6J male mice raised in pathogen-free facilities, mice were housed at 22°C–25°C on a circadian 12-hour light/12-hour dark cycle (lights on at 7 am and off at 7 pm) with ad libitum access to food and water. All animals used in this study were male and were purchased from Guangdong Medical Laboratory Animal Center (Guangzhou, China). All the animal experimental protocols were approved by the Subcommittee on Research and Animal Care (SRAC) of Shenzhen institutes of advanced technology.

Note that full information on the approval of the study protocol must also be provided in the manuscript.

Clinical data

Policy information about clinical studies
All manuscripts should comply with the ICMJE guidelines for publication of clinical research and a completed CONSORT checklist must be included with all submissions.

Clinical trial registration	*Provide the trial registration number from ClinicalTrials.gov or an equivalent agency.*
Study protocol	*Note where the full trial protocol can be accessed OR if not available, explain why.*
Data collection	*Describe the settings and locales of data collection, noting the time periods of recruitment and data collection.*
Outcomes	*Describe how you pre-defined primary and secondary outcome measures and how you assessed these measures.*

Dual use research of concern

Policy information about dual use research of concern

Hazards

Could the accidental, deliberate or reckless misuse of agents or technologies generated in the work, or the application of information presented in the manuscript, pose a threat to:

No	Yes	
☐	☐	Public health
☐	☐	National security
☐	☐	Crops and/or livestock
☐	☐	Ecosystems
☐	☐	Any other significant area

Experiments of concern

Does the work involve any of these experiments of concern:

No	Yes	
☒	☐	Demonstrate how to render a vaccine ineffective
☒	☐	Confer resistance to therapeutically useful antibiotics or antiviral agents
☒	☐	Enhance the virulence of a pathogen or render a nonpathogen virulent
☒	☐	Increase transmissibility of a pathogen
☒	☐	Alter the host range of a pathogen
☒	☐	Enable evasion of diagnostic/detection modalities
☒	☐	Enable the weaponization of a biological agent or toxin
☒	☐	Any other potentially harmful combination of experiments and agents

ChIP-seq

Data deposition

☐ Confirm that both raw and final processed data have been deposited in a public database such as GEO.

☐ Confirm that you have deposited or provided access to graph files (e.g. BED files) for the called peaks.

Data access links<br>*May remain private before publication.*	*For "Initial submission" or "Revised version" documents, provide reviewer access links. For your "Final submission" document, provide a link to the deposited data.*
Files in database submission	*Provide a list of all files available in the database submission.*
Genome browser session<br>(e.g. UCSC)	*Provide a link to an anonymized genome browser session for "Initial submission" and "Revised version" documents only, to enable peer review. Write "no longer applicable" for "Final submission" documents.*

Methodology

Replicates	*Describe the experimental replicates, specifying number, type and replicate agreement.*
Sequencing depth	*Describe the sequencing depth for each experiment, providing the total number of reads, uniquely mapped reads, length of reads and whether they were paired- or single-end.*

Antibodies	*Describe the antibodies used for the ChIP-seq experiments; as applicable, provide supplier name, catalog number, clone name, and lot number.*
Peak calling parameters	*Specify the command line program and parameters used for read mapping and peak calling, including the ChIP, control and index files used.*
Data quality	*Describe the methods used to ensure data quality in full detail, including how many peaks are at FDR 5% and above 5-fold enrichment.*
Software	*Describe the software used to collect and analyze the ChIP-seq data. For custom code that has been deposited into a community repository, provide accession details.*

Flow Cytometry

Plots

Confirm that:

- [ ] The axis labels state the marker and fluorochrome used (e.g. CD4-FITC).
- [ ] The axis scales are clearly visible. Include numbers along axes only for bottom left plot of group (a 'group' is an analysis of identical markers).
- [ ] All plots are contour plots with outliers or pseudocolor plots.
- [ ] A numerical value for number of cells or percentage (with statistics) is provided.

Methodology

Sample preparation	*Describe the sample preparation, detailing the biological source of the cells and any tissue processing steps used.*
Instrument	*Identify the instrument used for data collection, specifying make and model number.*
Software	*Describe the software used to collect and analyze the flow cytometry data. For custom code that has been deposited into a community repository, provide accession details.*
Cell population abundance	*Describe the abundance of the relevant cell populations within post-sort fractions, providing details on the purity of the samples and how it was determined.*
Gating strategy	*Describe the gating strategy used for all relevant experiments, specifying the preliminary FSC/SSC gates of the starting cell population, indicating where boundaries between "positive" and "negative" staining cell populations are defined.*

- [ ] Tick this box to confirm that a figure exemplifying the gating strategy is provided in the Supplementary Information.

Magnetic resonance imaging

Experimental design

Design type	*Indicate task or resting state; event-related or block design.*
Design specifications	*Specify the number of blocks, trials or experimental units per session and/or subject, and specify the length of each trial or block (if trials are blocked) and interval between trials.*
Behavioral performance measures	*State number and/or type of variables recorded (e.g. correct button press, response time) and what statistics were used to establish that the subjects were performing the task as expected (e.g. mean, range, and/or standard deviation across subjects).*

Acquisition

Imaging type(s)	*Specify: functional, structural, diffusion, perfusion.*
Field strength	*Specify in Tesla*
Sequence & imaging parameters	*Specify the pulse sequence type (gradient echo, spin echo, etc.), imaging type (EPI, spiral, etc.), field of view, matrix size, slice thickness, orientation and TE/TR/flip angle.*
Area of acquisition	*State whether a whole brain scan was used OR define the area of acquisition, describing how the region was determined.*

Diffusion MRI ☐ Used ☐ Not used

Preprocessing

Preprocessing software	*Provide detail on software version and revision number and on specific parameters (model/functions, brain extraction, segmentation, smoothing kernel size, etc.).*
Normalization	*If data were normalized/standardized, describe the approach(es): specify linear or non-linear and define image types used for transformation OR indicate that data were not normalized and explain rationale for lack of normalization.*
Normalization template	*Describe the template used for normalization/transformation, specifying subject space or group standardized space (e.g. original Talairach, MNI305, ICBM152) OR indicate that the data were not normalized.*
Noise and artifact removal	*Describe your procedure(s) for artifact and structured noise removal, specifying motion parameters, tissue signals and physiological signals (heart rate, respiration).*
Volume censoring	*Define your software and/or method and criteria for volume censoring, and state the extent of such censoring.*

Statistical modeling & inference

Model type and settings	*Specify type (mass univariate, multivariate, RSA, predictive, etc.) and describe essential details of the model at the first and second levels (e.g. fixed, random or mixed effects; drift or auto-correlation).*
Effect(s) tested	*Define precise effect in terms of the task or stimulus conditions instead of psychological concepts and indicate whether ANOVA or factorial designs were used.*

Specify type of analysis: ☐ Whole brain ☐ ROI-based ☐ Both

Statistic type for inference<br>(See Eklund et al. 2016)	*Specify voxel-wise or cluster-wise and report all relevant parameters for cluster-wise methods.*
Correction	*Describe the type of correction and how it is obtained for multiple comparisons (e.g. FWE, FDR, permutation or Monte Carlo).*

Models & analysis

n/a	Involved in the study
☐	☐ Functional and/or effective connectivity
☐	☐ Graph analysis
☐	☐ Multivariate modeling or predictive analysis

Functional and/or effective connectivity	*Report the measures of dependence used and the model details (e.g. Pearson correlation, partial correlation, mutual information).*
Graph analysis	*Report the dependent variable and connectivity measure, specifying weighted graph or binarized graph, subject- or group-level, and the global and/or node summaries used (e.g. clustering coefficient, efficiency, etc.).*
Multivariate modeling and predictive analysis	*Specify independent variables, features extraction and dimension reduction, model, training and evaluation metrics.*
